# Screening and Identification of Therapeutic Targets for Pulmonary Arterial Hypertension Through Microarray Technology

**DOI:** 10.3389/fgene.2020.00782

**Published:** 2020-07-22

**Authors:** Qing Li, LingBing Meng, DePing Liu

**Affiliations:** ^1^Department of Cardiology, Beijing Hospital, National Center of Gerontology, Institute of Geriatric Medicine, Chinese Academy of Medical Sciences, Beijing, China; ^2^Departments of Cardiology, Peking Union Medical College, Chinese Academy of Medical Sciences, Beijing, China

**Keywords:** pulmonary arterial hypertension, cell proliferation, hub gene, microarray technology, differentially expressed genes

## Abstract

Pulmonary arterial hypertension (PAH) is a rare but fatal disease characterized by vascular cell proliferation; the pathogenesis of PAH has yet to be fully elucidated. Publicly available genetic data were downloaded from the Gene Expression Omnibus (GEO) database, and gene set enrichment analysis (GSEA) was used to determine significant differences in gene expression between tissues with PAH and healthy lung tissues. Differentially expressed genes (DEGs) were identified using the online tool, GEO2R, and functional annotation of DEGs was performed using Gene Ontology (GO) and the Kyoto Encyclopedia of Genes and Genomes (KEGG) analysis. Next, the construction and module analysis of the protein–protein interaction (PPI) network and verification of the expression level of hub genes was performed. Finally, prediction and enrichment analysis of microRNAs associated with the hub genes was carried out. A total of 110 DEGs were detected by screening PAH and healthy lung samples. The expression of nine genes [polo-like kinase 4 (PLK4), centromere protein U, kinesin family member 20B, structural maintenance of chromosome 2 (SMC2), abnormal spindle microtubule assembly, Fanconi Anemia complementation group I, kinesin family member 18A, spindle apparatus coiled-coil protein 1, and MIS18 binding protein 1] was elevated in PAH; this was statistically significant compared with their expression in healthy lung tissue, and they were identified as hub genes. GO and KEGG analysis showed that the variations in DEGs were abundant in DNA-templated transcription, sister chromatid cohesion, mitotic nuclear division, cell proliferation, and regulation of the actin cytoskeleton. In conclusion, this study has successfully identified hub genes and key pathways of PAH, with a total of 110 DEGs and nine hub genes related to PAH, especially the PLK4 and SMC2 genes, thus providing important clues for the in-depth understanding of the molecular mechanism of PAH and providing potential therapeutic targets.

## Introduction

Pulmonary arterial hypertension (PAH) refers to the elevation of pulmonary artery pressure in isolation while pressure in the left atrium and pulmonary vein is normal; the condition is mainly due to increased pulmonary vascular resistance caused by lesions in pulmonary arterioles themselves (Simonneau et al., 2019). The prevalence of PAH is 15–60 cases/million population/year, while the incidence is 5–10 cases/million population/year (Ling et al., 2012; Lau et al., 2017); the survival time of untreated patients following diagnosis is 5–7 years (Benza et al., 2012). Characteristic pathological changes associated with PAH are proliferative remodeling, including pulmonary artery medial hypertrophy, intimal concentric or eccentric proliferation and fibrosis, adventitia thickening and fibrosis, and perivascular inflammatory cell infiltration (Humbert et al., 2019).

The pathogenesis of PAH has not been fully elucidated, although its development is closely related to pulmonary vascular remodeling. Genetic factors, epigenetic factors, and environmental factors contribute to the dysregulation of a variety of vasoactive molecules and ion channels, which in turn activates a series of complex signaling pathways (Morrell et al., 2019; Napoli et al., 2019), leading to phenotypic abnormalities in vascular cells (Cai et al., 2018). Pulmonary artery endothelial cells (PAECs), pulmonary artery smooth muscle cells (PASMCs), and pulmonary artery adventitia fibroblasts (PAAFs) all have a proliferative, anti-apoptotic, carcinoid phenotype (Caruso et al., 2017; Pullamsetti et al., 2017; Zhang et al., 2017). Currently, the treatment of PAH can, through the use of endothelin receptor antagonists, phosphodiesterase inhibitors, and guanylate cyclase agonists, reduce pulmonary vascular resistance; primarily, this improves a patient’s symptoms but cannot completely reverse pulmonary vascular remodeling (Farber et al., 2015). Therefore, no specific anti-remodeling strategy for PAH has yet been approved, and we must improve our understanding of its pathological mechanisms to explore new therapeutic targets.

Gene-sequencing technology enables rapid, inexpensive sequencing of the entire coding region of a genome or an entire genome. The systematic analysis of gene expression profiles from the perspective of gene regulation is a powerful tool for revealing the pathogenesis of disease (Dumas et al., 2018). Dannewitz Prosseda et al. (2019) used small interfering RNA (siRNA) high-throughput screening of 20,000 genes in combination with a multi-cohort analysis of published PAH-RNA expression datasets and found that fragile histidine triad (FHIT) expression was down-regulated in patients with PAH. FHIT is involved in the apoptosis and proliferation of multiple cell types and may be associated with the abnormal proliferation phenotype of PAECs and PASMCs. Using a weighted gene co-expression network analysis and calculating module-trait correlations based on a public microarray dataset, Wang et al. (2018) found that the YWHAB gene plays a role in both mitosis signaling and cell cycle pathways and that its up-regulation is closely related to the progression of PAH. Therefore, the integration of clinical, genomic, transcriptomic, proteomic, and metabolomic data provides the best opportunity to identify key signaling pathways for PAH (Wirka et al., 2018). Microarray technology can be used to detect genome-wide gene expression differences between healthy and diseased samples, and this technique has been widely used to research the pathobiology of PAH (Hoffmann et al., 2016; Elinoff et al., 2020).

For the present study, four datasets (GSE15197, GSE53408, GSE113439, and GSE117261) were downloaded from the Gene Expression Omnibus (GEO), the sample sources of which were collected from the lung tissue of healthy patients and patients with PAH. The interactive web tool, GEO2R, was used to screen and identify differentially expressed genes (DEGs) by comparing samples from the GEO series (Barrett et al., 2013). Then, the biological processes (BP), and signaling pathways involved in the two groups of DEGs were analyzed using Gene Ontology (GO) and the Kyoto Encyclopedia of Genes and Genomes (KEGG) (Minoru et al., 2016; The Gene Ontology Consortium, 2019). Next, the expression levels of the hub genes were displayed and verified by constructing a protein–protein interaction (PPI) network and filtering the significant modules of this network (Szklarczyk et al., 2017). In addition, enrichment analysis was used to predict microRNAs associated with the hub genes. Finally, we analyzed chemicals that might affect the expression of the hub genes in patients with PAH. The purpose of this study was to identify potential therapeutic targets for PAH.

## Materials and Methods

### Microarray Data Acquisition

We downloaded four genetic datasets (GSE15197, GSE53408, GSE113439, and GSE117261) from the GEO.^1^ The GSE15197 dataset contained 26 samples from patients with PAH and 13 healthy lung tissue samples. The GSE53408 dataset consisted of 12 PAH tissue samples and 11 healthy lung tissue samples. The GSE113439 dataset consisted of 15 PAH tissue samples and 11 healthy lung tissue samples. The microarray dataset of GSE117261 comprised 58 samples of PAH tissue samples and 25 healthy tissue samples. First, we used GSE113439 to explore the possibility of gene expression differences between tissues from cases of PAH and healthy tissues. Then, GSE15197, GSE53408, and GSE113439 were used to identify key PAH-related genes, while GSE117261 was used to verify the expression of hub genes.

### Gene Set Enrichment Analysis

Gene set enrichment analysis (GSEA) was used to determine whether a gene set showed statistically significant, concordant differences between two biological states; this was an efficient computational method. GSEA software was downloaded from the Broad Institute website.^2^ GSEA analysis was conducted on the three datasets, respectively, and the results were obviously similar. The representative results of GSE113439 were selected for demonstration.

### Identification of DEGs

GEO2R^3^ is a useful tool for identifying DEGs in the GEO platform: it was used to identify DEGs between PAH samples and healthy lung tissues. We averaged the duplicate probe sets. |log2FC| > 1 and adj. *P*-value < 0.05 (Fold change = PAH sample expression/healthy sample expression) was set as the cutoff standard. Subsequently, we visualized the DEGs by drawing volcano plots and heatmaps using R software (version 3.5.3) and FunRich software (Functional Enrichment analysis tool, version 3.1.3). We also plotted a Venn diagram to show the intersections among the three datasets.

### Gene Ontology and KEGG Enrichment Analysis

Gene Ontology and KEGG enrichment analysis was performed using the Database for Annotation, Visualization and Integrated Discovery (DAVID, version 6.8), which is an online platform that can be used to elucidate potential biological meanings behind DEGs. We used the ggplot package in R (version 3.5.3) to visualize the results.

### PPI Network Construction and Module Analysis

The Search Tool for the Retrieval of Interacting Genes (STRING)^4^ database and Cytoscape software (version 3.7.1) were used to construct the PPI network. The Molecular Complex Detection (MCODE) tool in Cytoscape was applied to identify the significant modules. The parameters were set as follows: degree of cut off = 2, node score cutoff = 0.2, k-core = 2, and maximum depth = 100. We used R to produce heatmaps to show the expression levels of hub genes in the modules of the three datasets.

### Hub Gene Expression Level Validation

We used the GSE117261 dataset to verify the expression levels of the hub genes. Boxplots were produced to visualize the results, using GraphPad prism (version 8.0.2).

### Prediction and Enrichment Analysis of microRNAs Related to Hub Genes

We used TargetScan^5^ to predict microRNAs associated with hub genes; this is an open database that reveals potential biological relationships between microRNAs and genes. We also used DNA Intelligent Analysis (DIANA)-miRPath v3.0, an online enrichment analysis tool, to conduct an enrichment analysis of the predicted microRNAs.

### Identification of Chemicals Associated With Hub Genes

The Comparative Toxicogenomics Database (CTD)^6^ is a publicly available database designed to predict how environmental factors can affect human health. It provides information on chemical–gene/protein interactions, chemical–disease, and gene–disease relationships. These data can be used to help understand the relationships among genes, environmental factors, and diseases. We used this database to identify chemicals that might affect these hub genes and which have not previously been identified.

## Results

### Significant Differences in Gene Expression Between PAH Tissues and Healthy Lung Tissues Revealed by GSEA Analysis

**FIGURE 1:**
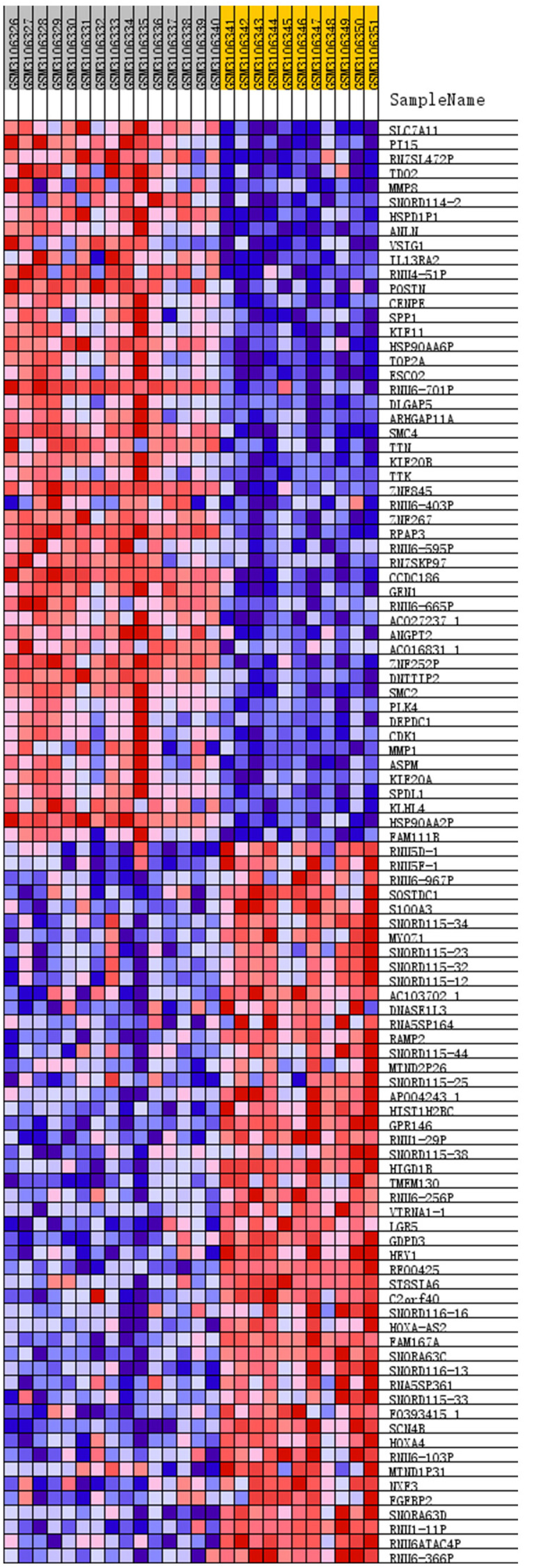
Heatmap of top 50 features for each phenotype. The PAH group: GSM3106326 – GSM3106340, and the normal lung tissue group: GSM3106341 – GSM3106351. Removed duplicate probes in GSE113439 and identified a total of 23,410 genes. Among these, 5,525 genes showed differences in expression between PAH tissues and healthy lung tissues. The expression of 3,964 genes was increased in PAH tissues, while 1,291 genes were upregulated in healthy lung tissues. The heatmap showed the two groups of genes that were in the top 50 for upregulation or downregulation. Red indicates that the gene expression is up-regulated, while blue indicates that the gene expression is down-regulated. In the same color, the darker the color, the more significant it was.

**FIGURE 2:**
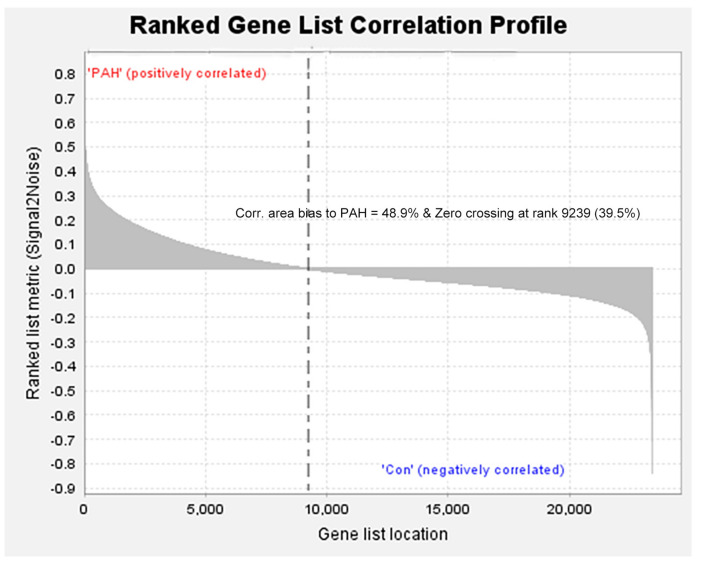
All genes were distributed according to the signal-to-noise ratio (SNR). Based on its SNR, the distribution of 23,410 genes in the two groups was mapped.

**FIGURE 3:**
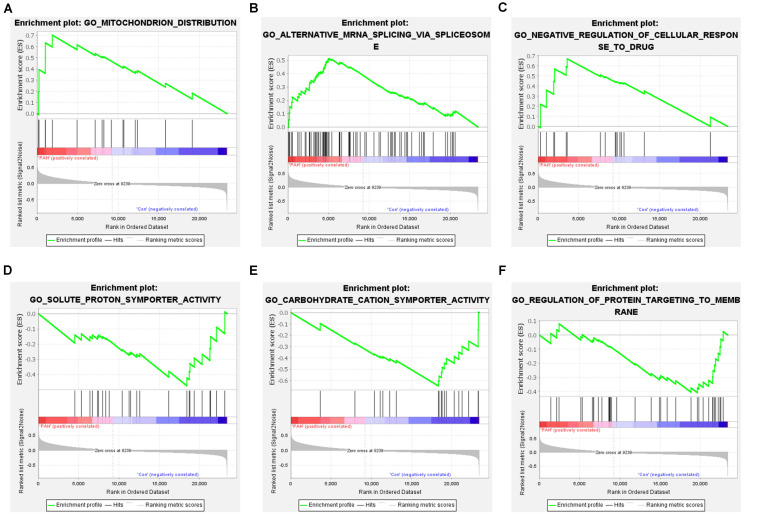
Gene set enrichment analysis of all genes. **(A–C)** Enrichment results of up-regulated genes. The GO terms enriched in PAH tissues included mitochondria distribution, alternative mRNA splicing via spliceosomes, and negative regulation of cellular responses to drugs. **(D–F)** Enrichment results of down-regulated genes. Solute proton symporter activity, carbohydrate cation symporter activity, and regulation of protein targeting to the membrane were mainly enriched in healthy lung tissues.

We removed duplicate probes in GSE113439 and identified a total of 23,410 genes. Among these, 5,525 genes showed differences in expression between PAH tissues and healthy lung tissues. The expression of 3,964 genes was increased in PAH tissues, while 1,291 genes were up-regulated in healthy lung tissues. We produced a heatmap to show the two groups of genes that were in the top 50 for up-regulation or down-regulation (Figure 1). At the same time, we mapped the distribution of 23,410 genes in the two groups according to their signal-to-noise ratio (SNR) (Figure 2). The GO terms enriched in PAH tissues included mitochondria distribution, alternative mRNA splicing via spliceosomes, and negative regulation of cellular responses to drugs. However, solute proton symporter activity, carbohydrate cation symporter activity, and regulation of protein targeting to the membrane were mainly enriched in healthy lung tissues (Figure 3). This showed that there were significant genetic differences between pulmonary hypertension tissue and healthy lung tissue.

### Identification of DEGs in PAH Tissues

**FIGURE 4:**
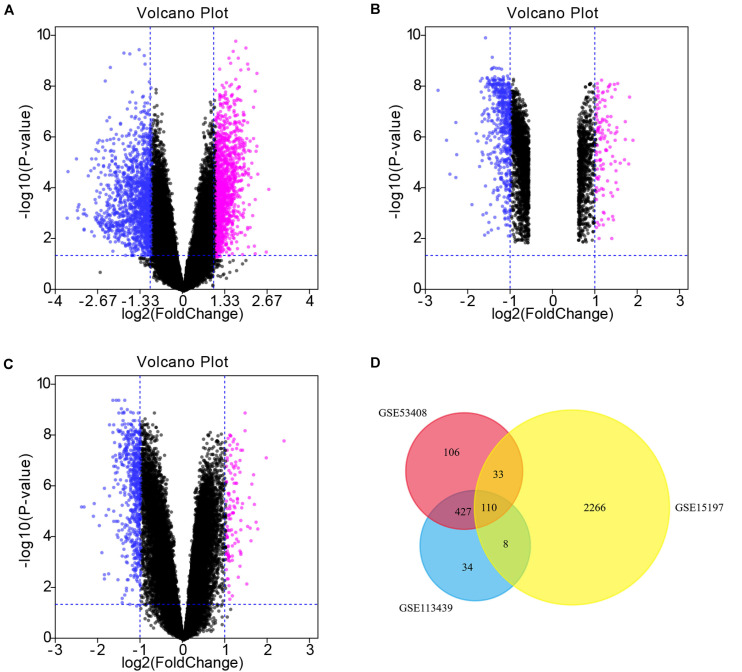
Volcano plots and Venn diagram. **(A–C)** DEGs were selected with —log2FC— > 1 and adj. *P*-value < 0.05 among the mRNA expression profiling sets GSE15197 **(A)**, GSE53408 **(B)**, GSE113439 **(C)**. Each symbol represents a different gene in the volcano plot. Pink symbols represent upregulated genes; blue symbols represent downregulated genes. **(D)** There were 676, 579, and 2417 DEGs obtained from the GSE53408, GSE113439, and GSE15197 datasets, respectively. The 3 datasets showed an overlap of 110 genes in the Venn diagram.

There were 676, 579, and 2417 DEGs obtained from the GSE53408, GSE113439, and GSE15197 datasets, respectively, which were downloaded from the GEO database (Table 1). We obtained 110 common DEGs, including 65 up-regulated genes and 45 down-regulated genes, from the intersection of the three DEG data sets (Table 2). The results were visualized in the form of volcano plots and a heatmap (Figure 4).

**TABLE 1:** Summary of pulmonary arterial hypertension datasets.

Series	Platform	GeneChip	Samples	DEGs
GSE53408	GPL6244	Affymetrix human gene 1.0 ST array	23	676
GSE15197	GPL6480	Agilent-014850 whole human genome microarray 4 × 44K G4112F	39	2417
GSE113439	GPL6244	Affymetrix human gene 1.0 ST array	26	579

**TABLE 2:** Screening of differentially expressed genes (DEGs) in pulmonary arterial hypertension.

DEGs	List of gene symbols
Up-regulated DEGs	ACSL4, ADAMTS9, ASPM, ATP6V1C1, ATRX, BCLAF1, BLZF1, BZW1, CCP110, CENPU, CMAHP, DDX21, DOCK10, EEA1, FANCI, FGF7, FRK, GAPT, GCC2, HOOK3, IARS, ITPR2, JMJD1C, KIAA0368, KIF18A, KIF20B, KRR1, MAPK6, MET, MIS18BP1, NAA15, PBRM1, PDGFD, PHF20L1, PIK3C2A, PLK4, POLK, PPP1R12A, PYROXD1, RANBP6, RIOK2, RNF6, S100A3, SAMD9, SCN4B, SERPINB2, SLC6A14, SLC7A11, SLK, SLTM, SMC2, SMC5, SOCS4, SOSTDC1, SPDL1, SRFBP1, STAG2, STEAP1, SUCLA2, THOC2, TXNRD1, USP34, ZC3H15, ZNF267, ZNF845
Down-regulated DEGs	ALAS2, ANKRD50, ASCC3, BOD1L1, CA1, CCDC186, DDX52, DST, EHF, EPHA4, ESF1, FMO5, FNBP1L, HIST2H2AC, HLTF, HOOK1, HSP90AA1, IGF1, IQGAP2, ITGA2, KIAA1109, LBH, MACC1, MYCBP2, MYO5A, N4BP2, NCL, NIPBL, NSF, OSBPL8, RFC1, RSPO3, RSRC1, SECISBP2L, SUPT16H, TFEC, TTN, TXLNG, UBXN4, USP15, VEPH1, ZC3H13, ZFX, ZNF148, ZNF292

### GO and KEGG Enrichment Analysis for DEGs

**FIGURE 5:**
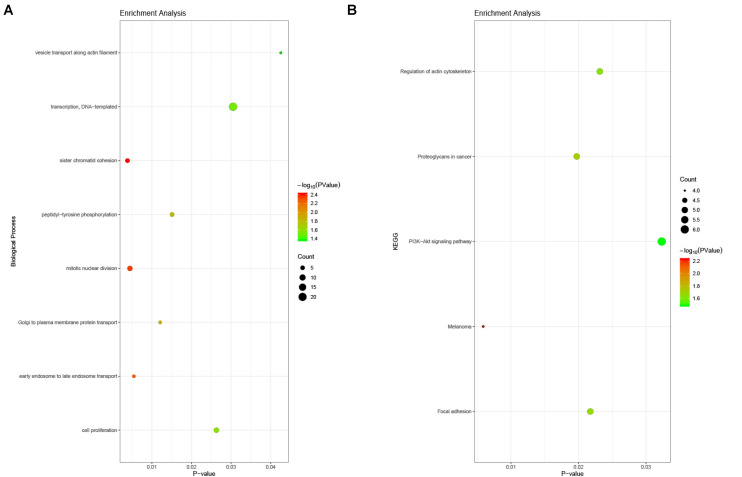
Enrichment analysis of DEGs in PAH. **(A)** Biological process analysis. The biological processes consisted mainly of DNA-templated transcription, sister chromatid cohesion, mitotic nuclear division, cell proliferation, peptidyl-tyrosine phosphorylation, Golgi to plasma membrane protein transport, and so on. **(B)** KEGG pathway analysis. The regulation of the actin cytoskeleton, the PI3K-Akt signaling pathway, and focal adhesion were noted.

The enrichment analysis for DEGs showed that the BP consisted mainly of DNA-templated transcription, sister chromatid cohesion, mitotic nuclear division, cell proliferation, peptidyl-tyrosine phosphorylation, Golgi to plasma membrane protein transport, and so on. With respect to the KEGG pathway, the regulation of the actin cytoskeleton, the PI3K-Akt signaling pathway, and focal adhesion were noted (Figure 5).

### Construction and Module Analysis of the PPI Network

**FIGURE 6:**
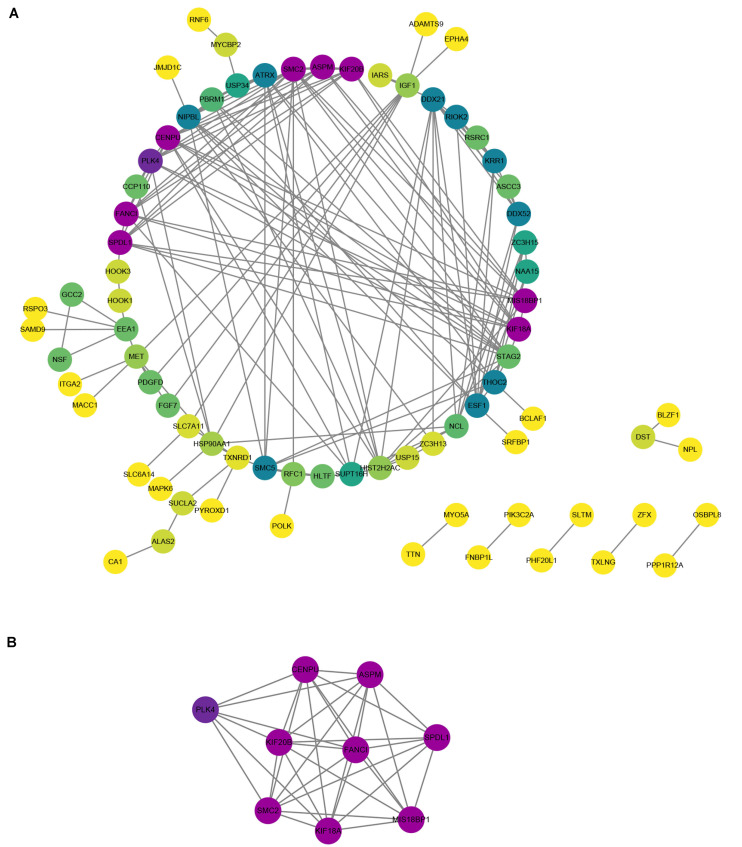
Protein–protein interaction (PPI) network and hub genes in PAH. **(A)** The PPI network of DEGs contained 77 nodes and 155 edges. Purple was the most significant, followed by green, and yellow was the least significant. In the same color, the darker the color, the more significant it was. **(B)** The most significant module was obtained from PPI network of DEGs.

To understand the relationships among DEGs, a PPI network was constructed, consisting of 77 nodes and 155 edges (Figure 6A). Module analysis was subsequently performed and the most significant module in the PPI network was identified. The results showed that the top nine candidate hub genes obtained were polo-like kinase 4 (PLK4), CENPU, KIF20B, structural maintenance of chromosome 2 (SMC2), ASPM, FANCI, KIF18A, SPDL1, and MIS18BP1 (Figure 6B).

### Hub Gene Expression Level Validation

**FIGURE 7:**
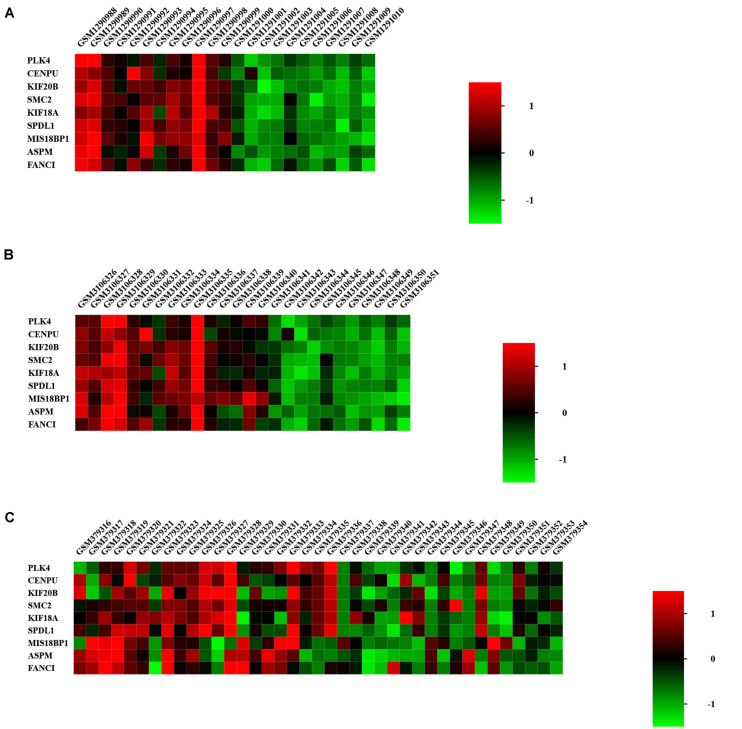
Heatmap of hub genes in GSE53408 **(A)**, GSE113439 **(B)**, and GSE15197 **(C)**. The nine identified hub genes showed elevated expression in the PAH group.

**FIGURE 8:**
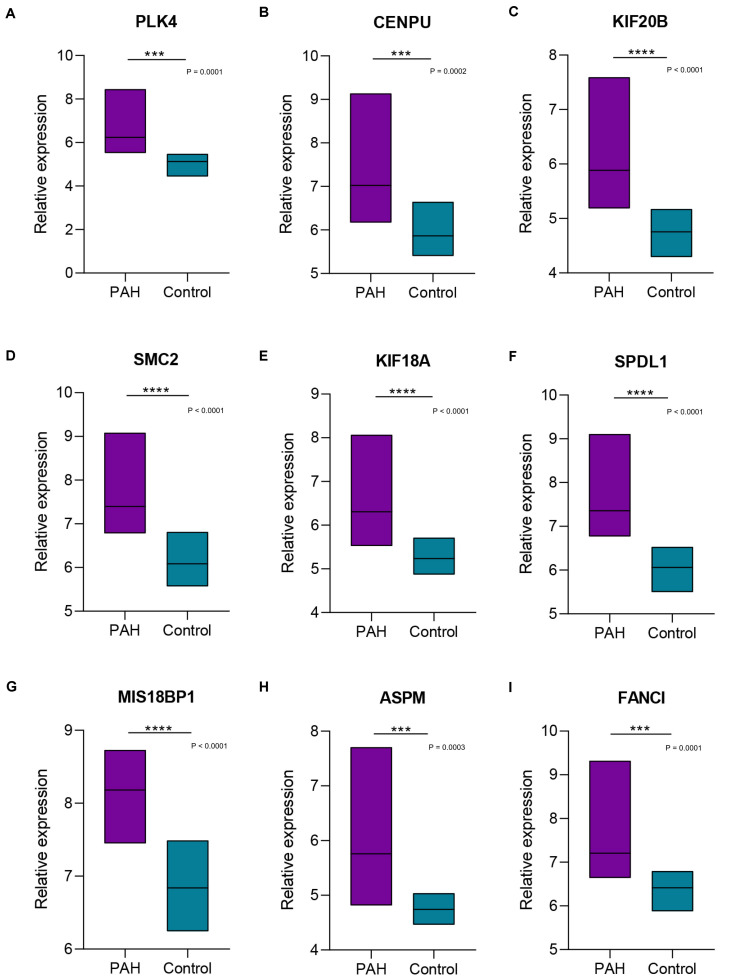
Boxplots of the expression levels of nine hub genes in GSE117261. The expression of the nine key genes [PLK4 **(A)**, CENPU **(B)**, KIF20B **(C)**, SMC2 **(D)**, KIF18A **(E)**, SPDL1 **(F)**, MIS18BP1 **(G)**, ASPM **(H)**, FANCI **(I)**] was statistically significantly elevated in pulmonary arterial hypertension tissues compared with their expression in healthy lung tissues. ***, **** Indicate significant correlations at *P* < 0.001, *P* < 0.0001, respectively.

We focused on the expression levels of the nine hub genes in the GSE15197, GSE53408, and GSE113439 datasets and plotted heatmaps for them (Figure 7). The results showed that the nine identified hub genes showed elevated expression in the PAH group. We verified their expression again in the GSE117261 dataset, and the results indicated that the expression of these nine key genes was statistically significantly elevated in PAH tissues compared with their expression in healthy lung tissues (Figure 8).

### Prediction and Enrichment Analysis of microRNAs Related to Hub Genes

**FIGURE 9:**
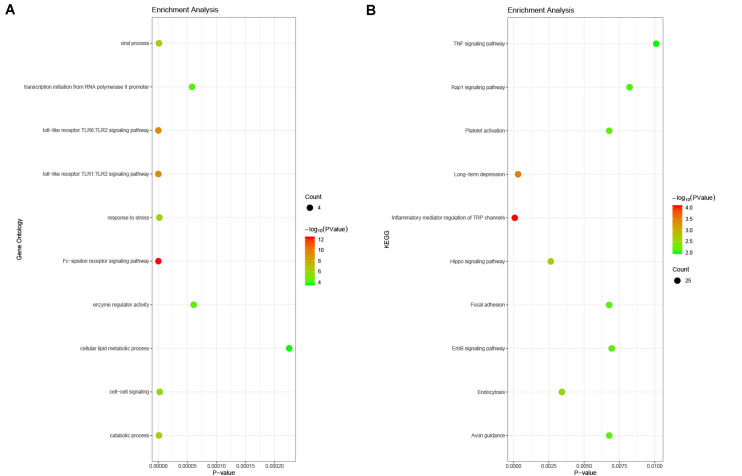
Enrichment analysis of the microRNAs related to hub genes. **(A)** Gene ontology analysis. The microRNAs related to hub genes were abundant in the toll-like receptor TLR1:TLR2 signaling pathway, viral processes, and the Fc-epsilon receptor signaling pathway. **(B)** KEGG enrichment analysis. These miRNAs were mainly enriched in the TNF signaling pathway, focal adhesion, and platelet activation.

In order to understand the mechanism and regulatory network of the hub genes, microRNAs related to the hub genes were predicted and analyzed by enrichment analysis (Table 3). GO analysis showed that these miRNAs were abundant in the toll-like receptor TLR1:TLR2 signaling pathway, viral processes, and the Fc-epsilon receptor signaling pathway. In the KEGG pathway enrichment analysis, they were mainly enriched in the TNF signaling pathway, focal adhesion, and platelet activation (Figure 9).

**TABLE 3:** The potential microRNAs associated with the hub genes.

	Gene	Predicted microRNAs		Gene	Predicted microRNAs
1	PLK4	hsa-miR-4787-5p	6	FANCI	hsa-miR-212-5p
		hsa-miR-34c-3p			hsa-miR-645
		hsa-miR-1486-3p			hsa-miR-7706
2	CENPU	hsa-miR-599	7	KIF18A	hsa-miR-493-5p
		hsa-miR-496.2			hsa-miR-193a-5p
		hsa-miR-5582-5p			hsa-miR-6508-3p
3	KIF20B	hsa-miR-19b-3p	8	SPDL1	hsa-miR-141-3p
		hsa-miR-19a-3p			hsa-miR-200a-3p
		hsa-miR-5590-3p			hsa-miR-4477b
4	SMC2	hsa-miR-133a-3p.2	9	MIS18BP1	hsa-miR-30e-5p
		hsa-miR-133b			hsa-miR-30d-5p
		hsa-miR-383-5p.1			hsa-miR-30a-5p
5	ASPM	hsa-miR-1284			
		hsa-miR-4307			
		hsa-miR-5739			

### Identification of Chemicals Associated With Hub Genes

We analyzed each of the hub genes and predicted the chemicals that might affect their expression in PAH. For instance, copper sulfate, epigallocatechin gallate, oxygen, quercetin, and valproic acid may affect the expression of KIF20B. The rest are detailed in Table 4.

**TABLE 4:** Chemicals that may affect the expression of hub genes in pulmonary arterial hypertension.

Num.	Gene ID	Gene symbol	Gene name	Inference network	Inference score
1	9585	KIF20B	Kinesin Family Member 20B	Copper sulfate, epigallocatechin gallate, oxygen, quercetin, valproic acid	12.78
2	10592	SMC2	Structural maintenance Of chromosomes 2	4,4′-diaminodiphenylmethane, epigallocatechin gallate, quercetin, resveratrol, valproic acid	12.74
3	10733	PLK4	Polo like kinase 4	(+)-JQ1 compound, oxygen, paclitaxel, paraquat, resveratrol, silicon dioxide, valproic acid, vorinostat	22.45
4	54908	SPDL1	Spindle apparatus coiled-coil protein 1	Copper sulfate, methamphetamine, paraquat, resveratrol, smoke, valproic acid	3.85
5	55215	FANCI	Fanconi anemia complementation group I	Cobaltous chloride	3.83
6	55320	MIS18BP1	MIS18 binding protein 1	Copper sulfate, quercetin, resveratrol, valproic acid, vorinostat	15.25
7	79682	CENPU	Centromere protein U	Indomethacin	3.84
8	81930	KIF18A	Kinesin Family Member 18A	Amphetamine, copper Sulfate, epigallocatechin gallate, (+)-JQ1 compound, quercetin, resveratrol, silicon dioxide, valproic acid	24.22
9	259266	ASPM	Abnormal Spindle microtubule assembly	Cobaltous chloride	3.61

## Discussion

Mitotic fission is increased in PAH, which is a hyperproliferative, apoptosis-resistant disease; pulmonary artery remodeling caused by the imbalance between proliferation and apoptosis in vascular walls is an important characteristic of PAH. Although the understanding of the pathophysiology of PAH has greatly improved in recent years, there remains an urgent need to fully understand the internal mechanism that drive vascular remodeling. The abnormal proliferation and cell cycle dysregulation of pulmonary vascular cells in PAH involves a complex process. Current treatment for PAH is dependent on pulmonary vasodilators, which cannot inhibit the underlying mechanism of vascular proliferative remodeling (Spiekerkoetter et al., 2019). Therefore, it is imperative to explore the molecular mechanisms linked to the occurrence and development of PAH for the early diagnosis, treatment, and prognosis of this condition and to determine effective anti-proliferation/pro-apoptosis strategies that can block the disease process (Vaillancourt et al., 2015).

Microarray technology can simultaneously express a large number of genes efficiently and accurately. It can also be used to conduct gene expression profiling to obtain information relating to gene function and the regulation of gene expression, providing a potential tool for the further exploration of the expression of hub genes, and the regulatory networks involved in the pathogenesis of PAH. Kikuchi et al. (2018) performed microarray analyses using PAH-PASMCs and found that, compared with the genes in control PASMCs, 1,858 genes had significantly changed (*P* < 0.05), of which selenoprotein P (SeP) was up-regulated 32 times compared with its expression in control PASMCs. SeP in PASMCs promotes cell proliferation in an autocrine/paracrine manner by increasing oxidative stress and mitochondrial dysfunction. In the present study, three data sets (GSE15197, GSE53408, and GSE113439) were screened for DEGs; there were 110 DEGs discovered, which shared nine hub genes in common: PLK4, CENPU, KIF20B, SMC2, ASPM, FANCI, KIF18A, SPDL1, and MIS18BP1. Microarray and bioinformatics analyses emphasized that the main gene expression changes in PAH cells occur in genes relating to the regulation of cell cycle progression and proliferation. Among them, PLK4 and SMC2 were differentially expressed and showed better homogeneity between samples of PAH and samples of the Control group. A literature search performed using PubMed showed that studies into the role played by PLK4 in PAH have so far been limited. The results of the present study might represent a good starting point for subsequent investigations. We suggest that these two genes (PLK4 and SMC2) are significant and should be investigated further.

Polo-like kinase 4 is a highly conserved serine/threonine protein kinase, belonging to the polo-like protein kinase family. As a self-regulating cell cycle regulator protein, PLK4 plays a pivotal role in the process of centriole duplication (Moyer et al., 2015). Centriole duplication is strictly controlled in the body, since it is essential for successful chromosome segregation and maintenance of genomic stability; abnormalities in this process are associated with a variety of human diseases, including tumors (Godinho et al., 2014; Levine et al., 2017). PLK4 has been studied as a potential therapeutic target in tumors for many years, as it is the key regulator of centriole biogenesis. In proliferating tissues, PLK4 is expressed in the form of low-abundance enzymes under normal conditions and it phosphorylates itself for destruction; this self-regulating disruption helps to limit centriole duplication to once per cell cycle by controlling the level of endogenous PLK4 (Maniswami et al., 2018). Phase separation of PLK4 drives centriole biogenesis through autoactivation and clustering (Park et al., 2019). The intrinsic self-organization of PLK4 is related to the symmetrical breaking during the process of centriole duplication, and the formation of its spatial pattern is the determinant of the replication site of the parent centriole (Yamamoto and Kitagawa, 2019). PLK4 can function in a homeostatic manner to balance the growth rate and the growth period to determine the final centriole size (Gemble and Basto, 2018). PLK4 is expressed mainly in actively dividing tissues and cells; its abnormal expression level can affect the normal duplication of centrioles, causing changes in the number of centrioles and abnormalities in centrosome structure. Low levels of PLK4 activity can damage the duplication of centrioles, while high levels of PLK4 activity can lead to excessive duplication of centrioles, thus interfering with the normal process of mitosis, which is closely related to the occurrence and development of abnormal cell proliferation (Liu et al., 2018).

Wong et al. (2015) believed that centrioles are essential for the proliferation of healthy human cells and that inhibition of PLK4 prevents the assembly of centrioles. Mason et al. (2014) used a systematic approach of RNAi screening in combination with gene expression analysis and demonstrated experimentally that the inhibition of PLK4 activity can result in the dysregulation of mitochondria, mitotic defects, and cell death in tumor cells, as well as significantly inhibiting the growth of breast cancer xenograft tumor models. It is worth noting that p53 can indirectly inhibit the transcription of PLK4 cell cycle genes through the p53–p21–DREAM–CDE/CHR pathway, leading to cell cycle arrest or apoptosis (Fischer et al., 2016). PLK4 is located in different subcellular organelles depending on the stage of the cell cycle, including centrosomes, centromeres, cleavage grooves, and mesosomes. Press et al. (2019) used immunofluorescence to confirm that PLK4 is located in the centrosome during S phase, plays a key regulatory role in centrosome replication during cell division, and may function as an integrin. Inhibition of PLK4 kinase activity can prevent cell division, causing polyploidy and mitotic disorders.

Polo-like kinase 4-mediated centriole duplication plays a crucial role in maintaining correct mitosis in healthy cells, while its deregulation can cause abnormal centrosome numbers, mitotic defects, and chromosomal instability, resulting in abnormal cell proliferation (Kazazian et al., 2016; Kawakami et al., 2018). Our study combined four different microarray datasets, which we analyzed and found that there was increased PLK4 expression in the lung tissue of patients with PAH. The overexpression of PLK4 can affect cell centriole duplication, which may lead to changes in the number of centrioles and abnormalities in centrosome structure, such that vascular wall cells have a greater tendency to divide and proliferate, which is related to the proliferation-antiapoptotic biological behavior of PAH seen clinically. Therefore, the role of PLK4 in the cell proliferative response of PAH deserves further research, and it may be of particular significance to explore the expression of PLK4 in relation to the diagnosis and prognostic evaluation of PAH.

The structural maintenance of chromosome 2 gene is a member of the ATPase family that is mainly involved in mitotic cell division and the assembly process of genomic DNA. The SMC2 protein product belongs to the condensin complex and plays a crucial role in the packaging of chromatin prior to cell division, which is necessary for the correct separation of chromosomes and the maintenance of chromosome stability. SMC2 promotes the compression and dissociation of sister chromatids, enabling them to correctly dissociate into daughter cells during anaphase of cell division (Eeftens et al., 2016). By combining a meta-analysis with the largest genome-wide association (GWA) meta-analysis datasets, Kar et al. (2016) found that SMC2 is associated with susceptibility to breast and ovarian cancer. Feng et al. (2019) used genome-wide association studies (GWASs) of pancreatic cancer datasets and found that the expression level of SMC2 mRNA in human pancreatic cancer tissues was significantly higher than that in adjacent non-neoplastic pancreatic tissues. Davalos et al. (2012) found that SMC2 is a direct transcriptional target of the WNT signaling pathway, and experimental results showed that the down-regulation of SMC2 expression can inhibit WNT-activated cell proliferation; therefore, SMC2 could be used as a novel target for therapeutic interventions for tumors. SMC2, which ensures cell mitosis and rapid proliferation, has to date primarily been studied in tumors and, although its role in PAH has not been determined, increased SMC2 expression has been observed in PAH, as shown in the present study. In addition, we used TargetScan to predict and found that miR-133a-3p.2, miR-133b, and miR-383-5p.1 are SMC2-related microRNAs. Among them, Legchenko et al. (2018) found that miR-133b expression was down-regulated in peripheral pulmonary arteries and up-regulated in plexiform vascular lesions, which is likely dependent on the global and segmental disease stage. The role of SMC2 in both cell proliferation and in PAH remains to be clarified.

The work described here involved rigorous bioinformatics analyses, but some limitations remained, specifically that the results of this study were based on a computer analysis, and subsequent *in vivo* and *in vitro* validation is necessary.

## Conclusion

In conclusion, four datasets from the GEO database were integrated with bioinformatics technologies for analysis. A total of 110 DEGs were obtained, with nine hub genes related to PAH (PLK4, CENPU, KIF20B, SMC2, ASPM, FANCI, KIF18A, SPDL1, and MIS18BP1), of which PLK4 and SMC2 were particularly significant. It is worth noting that there have been no previous experimental studies performed to investigate the association of PLK4 and SMC2 with PAH. Therefore, the findings of this study suggest that more investigation and research should be conducted in this area to develop new ideas and therapeutic targets for the diagnosis and treatment of PAH.

## Data Availability Statement

The datasets generated for this study can be found in the Gene Expression Omnibus (GEO, www.ncbi.nlm.nih.gov/geo), GSE15197, GSE53408, GSE113439, and GSE117261.

## Author Contributions

QL and LM conceived and designed the study, collected the datasets, analyzed the data, and designed the draft of the research process. QL was the major contributor to writing and submitting the manuscript. DL was involved in critically revising the manuscript for important intellectual content. All authors read and approved the final manuscript prior to submission.

## Conflict of Interest

The authors declare that the research was conducted in the absence of any commercial or financial relationships that could be construed as a potential conflict of interest.

## Footnotes

1 www.ncbi.nlm.nih.gov/geo

2 http://www.broadinstitute.org/gsea/index.jsp

3 https://www.ncbi.nlm.nih.gov/geo/geo2r/

4 https://string-db.org

5 www.targetscan.org

6 http://ctdbase.org
